# Supplementary material for: Muscle Activation in Traditional and Experimental Barbell Bench Press Exercise: A Potential New Tool for Fitness Maintenance
Source: Sports (Basel). 2019 Oct 17;7(10):224. doi: 10.3390/sports7100224 (PMC6835758; doi:10.3390/sports7100224)
Supplement: Supplementary file 1 [file sports-07-00224-s001.pdf]

sports

Article

Muscle Activation in Traditional and Experimental Barbell Bench Press Exercise: A Potential New Tool for Fitness Maintenance

Andrea Melani [^,1], Giuliana Gobbi [^,2,3], Daniela Galli [*,2,3], Cecilia Carubbi [3], Elena Masselli [3], Luca Maria Neri [4], Gaspare Giovinco [5], Antonio Cicchella [6], Laura Galuppo [7], Valentina Presta [7], Mauro Vaccarezza [8,9], Marco Vitale [2,3] and Prisco Mirandola [2,3]

1 PhD program in "Systems, technologies and devices for movement and health" at the University of Cassino and Southern Lazio, Cassino 03043,, Italy; andreamelani@gmail.com
2 Sport and Exercise Medicine Centre (SEM), Department of Medicine and Surgery, University of Parma, 43125 Parma, Italy; giuliana.gobbi@unipr.it (G.G.); marco.vitale@unipr.it (M.V.); prisco.mirandola@unipr.it (P.M.)
3 Unit SBiBiT (Biomedical, Biotecnological and Translational Sciences), Department of Medicine and Surgery, University of Parma, 43125 Parma, Italy; cecilia.carubbi@unipr.it (C.C.); elena.masselli@unipr.it (E.M.)
4 Anatomy and Histology section, Department of Morphology and Experimental Medicine and Surgery, University of Ferrara, 44121 Ferrara, Italy; Lumane265@gmail.com
5 Department of Civil and Mechanical Engineering, University of Cassino and Southern Lazio, Cassino 03043, Italy; andya8380@gmail.com
6 Department for Life Quality Studies, University of Bologna, Bologna 40126, Italy; antonio.cicchella@unibo.it
7 PhD program in "Molecular Medicine" at the University of Parma, 43125 Parma, Italy; laura.galuppo@studenti.unipr.it (L.G.); valentina.presta@unipr.it (V.P.)
8 Department of Human, Social and Health Sciences, University of Cassino and Southern Lazio, Cassino 03043, Italy; mauro.vaccarezza@curtin.edu.au
9 School of Pharmacy and Biomedical Sciences, Faculty of Health Sciences, Curtin University, Bentley, Perth 6102, Western Australia, Australia.
* Correspondence: daniela.galli@unipr.it; Tel.: +39 0521 906306; Fax: +39 0521 033039
^ Equally contributed, first author

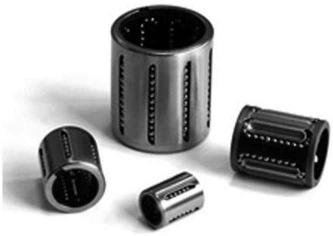

Figure S1. The anti-rolling device of the handles. Ball bearings embedded in semicircular milling in the longitudinal direction.

© 2019 by the authors. Submitted for possible open access publication under the terms and conditions of the Creative Commons Attribution (CC BY) license (http://creativecommons.org/licenses/by/4.0/).
